# Pentagalloyl Glucose from *Bouea macrophylla* Suppresses the Epithelial–Mesenchymal Transition and Synergizes the Doxorubicin-Induced Anticancer and Anti-Migration Effects in Triple-Negative Breast Cancer

**DOI:** 10.3390/ph17121729

**Published:** 2024-12-20

**Authors:** Jiraporn Kantapan, Phattarawadee Innuan, Sarawut Kongkarnka, Padchanee Sangthong, Nathupakorn Dechsupa

**Affiliations:** 1 Molecular Imaging and Therapy Research Unit, Department of Radiologic Technology, Faculty of Associated Medical Sciences, Chiang Mai University, Chiang Mai 50200, Thailand; jiraporn.kan@cmu.ac.th (J.K.); phattarawadeeinnuan@gmail.com (P.I.); 2 Department of Radiologic Technology, Faculty of Associated Medical Sciences, Chiang Mai University, Chiang Mai 50200, Thailand; 3 Department of Pathology, Faculty of Medicine, Chiang Mai University, Chiang Mai 50200, Thailand; srawutzi@gmail.com; 4 Division of Biochemistry and Biochemical Innovation, Department of Chemistry, Faculty of Science, Chiang Mai University, Chiang Mai 50200, Thailand; padchanee.sangthong@cmu.ac.th

**Keywords:** pentagalloyl glucose (PGG), triple-negative breast cancer (TNBC), epithelial–mesenchymal transition (EMT), doxorubicin, synergistic

## Abstract

**Background:** Triple-negative breast cancer (TNBC) represents an aggressive form of breast cancer with few available therapeutic options. Chemotherapy, particularly with drugs like doxorubicin (DOX), remains the cornerstone of treatment for this challenging subtype. However, the clinical utility of DOX is hampered by adverse effects that escalate with higher doses and drug resistance, underscoring the need for alternative therapies. This study explored the efficacy of pentagalloyl glucose (PGG), a natural polyphenol derived from *Bouea macrophylla*, in enhancing DOX’s anticancer effects and suppressing the epithelial–mesenchymal transition (EMT) in TNBC cells. **Methods:** This study employed diverse methodologies to assess the effects of PGG and DOX on TNBC cells. MDA-MB231 triple-negative breast cancer cells were used to evaluate cell viability, migration, invasion, apoptosis, mitochondrial membrane potential, and protein expression through techniques including MTT assays, wound healing assays, flow cytometry, Western blotting, and immunofluorescence**. Results:** Our findings demonstrate that PGG combined with DOX significantly inhibits TNBC cell proliferation, migration, and invasion. PGG enhances DOX-induced apoptosis by disrupting the mitochondrial membrane potential and activating caspase pathways; consequently, the activation of caspase-3 and the cleavage of PARP are increased. Additionally, the study shows that the combination treatment upregulates ERK signaling, further promoting apoptosis. Moreover, PGG reverses DOX-induced EMT by downregulating mesenchymal markers (vimentin and β-catenin) and upregulating epithelial markers (E-cadherin). Furthermore, it effectively inhibits STAT3 phosphorylation, associated with cell survival and migration. **Conclusions:** These results highlight the potential of PGG as an adjuvant therapy in TNBC treatment. PGG synergizes with DOX, which potentiates its anticancer effects while mitigating adverse reactions.

## 1. Introduction

Triple-negative breast cancer (TNBC) is a particularly aggressive subtype of breast cancer, comprising approximately 15% of all breast cancer cases. TNBC is characterized by a lack of hormonal receptors and the amplification of HER2 amplicons, which limit treatment options. Thus, the current treatment strategies for TNBC patients still involve chemotherapy [1,2]. Due to its aggressive nature and limited therapy options, TNBC patients are highly likely to experience rapid recurrence, drug resistance, and, eventually, metastases [3,4,5]. As a result, new effective treatments against TNBC are urgently needed. Despite the fact that doxorubicin (DOX) is a widely used chemotherapeutic agent for effectively treating triple-negative breast cancer (TNBC), its clinical utility is constrained by dose-related undesirable side effects and the development of drug resistance [6,7]. Consequently, the development of effective targeted therapies for TNBC is a critical priority to enhance treatment outcomes.

The epithelial-to-mesenchymal transition (EMT) is a biological process in which epithelial cells transform and adopt invasive traits characteristic of mesenchymal cells. EMT has garnered attention from cancer researchers due to its strong association with the metastasis of cancer cells, as well as its correlation with cancer progression, cancer stem cells, invasion, and therapy resistance [8,9]. A defining feature of the epithelial-to-mesenchymal transition (EMT) is the diminished expression of the E-cadherin protein at the point of cell–cell contact. This transformation is accompanied by an increased expression of mesenchymal markers, including N-cadherin, vimentin, and fibronectin. In addition, several transcription factors (TFs) that serve as repressors of the E-cadherin promoter, such as Twist, Zeb1, and Zeb2, the Snail/Slug family, and others, are implicated in the EMT [10,11]. Recent findings indicate intricate connections between cells undergoing EMT and the emergence of drug resistance in tumors. Crucial investigations utilizing tumor cell lines highlight the participation of EMT in resistance mechanisms activated by radiotherapy and chemotherapy [12,13,14]. Furthermore, an enhanced capacity for cell invasion has been associated with resistance to chemotherapy. It has been reported that the upregulation of EMT-promoting transcription factors in breast cancer is the mechanism by which the development of drug resistance induces EMT [15,16]. As a result, EMT has been studied to comprehend the mechanism underlying cancer metastasis and the effects of anticancer drugs. Strategies involving combination therapy utilize new agents with different modes of action; the aim is to lower the concentration of the drug to a level of minimal concurring toxicity and to reduce the chance of drug resistance, which may impact the future development of more successful therapeutic regimens for TNBC [17]. Peptide–drug conjugates (PDCs) represent a promising strategy that combines the targeting specificity of peptides with the therapeutic potency of drugs. By using peptides as delivery vehicles, PDCs selectively direct cytotoxic drugs to cancer cells, thereby minimizing adverse effects and enhancing therapeutic efficacy [18,19]. However, PDCs face notable challenges, including limited in vivo stability due to the enzymatic degradation of peptides, difficulties in achieving efficient delivery to target tissues, and the complexity and costliness of production processes, which require precise chemical synthesis and purification. Additionally, potential immunogenicity and challenges in optimizing pharmacokinetics further complicate their clinical application [20,21]. Alternatively, combining chemotherapeutic drugs with bioactive compounds, such as polyphenols, presents a promising approach. Research has demonstrated that polyphenols can enhance the effects of cisplatin and doxorubicin, working synergistically to promote apoptosis in solid tumors [22,23,24]. Therefore, it is important to explore effective compounds derived from natural products that may work synergistically with doxorubicin activity and improve the therapeutic ratio.

Pentagalloyl glucose (PGG), also known as 1,2,3,4,6-penta-O-galloyl-beta-D-glucopyranoside, is a naturally occurring polyphenol and hydrolyzable tannin extracted from the seeds of *Bouea macrophylla*. PGG exhibits a broad spectrum of pharmacological properties, including anti-inflammatory, antiviral, and anticancer effects. Its anticancer activities include inhibiting cancer cell proliferation and DNA synthesis, inducing cell cycle arrest, promoting apoptosis in malignant cells, and reducing tumor angiogenesis and metastasis [25,26,27,28]. In our previous research, we validated that pretreatment with PGG extracted from *Bouea macrophylla* seed extract (MPSE) on breast cancer cells before radiation therapy effectively impedes the radiation-induced epithelial-to-mesenchymal transition (EMT) process. This process is associated with the radiation resistance of cancer cells. By impeding EMT, PGG enhances radiosensitivity. Intriguingly, PGG demonstrates synergistic effects in combination therapy [29], and such combined treatments exhibit comparable efficacy at lower concentrations for every single agent [30]. Furthermore, when PGG is combined with a chemotherapeutic drug, it mitigates the adverse reactions associated with a chemotherapeutic drug [31]. These attributes present significant advantages for the development of PGG combination therapy in the treatment of cancers. Hence, the primary objective of the current investigation was to ascertain whether PGG could effectively impede the epithelial-to-mesenchymal transition (EMT) process in triple-negative breast cancer cells. We hypothesized that combining PGG with the anticancer agent doxorubicin might yield synergistic benefits in treating triple-negative breast cancer (TNBC). Our research identified a synergistic effect between PGG and doxorubicin, manifested through the induction of disrupted mitochondrial membrane potential-mediated apoptosis. In addition, PGG mediated the inhibition of EMT markers and the invasion and migration of MDA-MB231 TNBC breast cancer cell lines. Our findings suggest that the amalgamation of PGG and doxorubicin holds promise as a prospective approach for the treatment of breast cancer.

## 2. Results

### 2.1. PGG Inhibits Cell Proliferation and Survival of Triple-Negative Breast Cancer Cells

**Figure 1:**
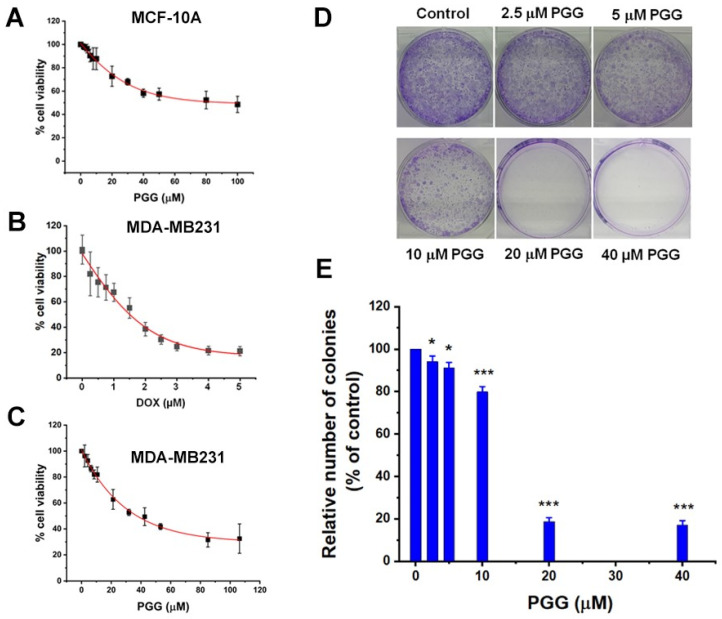
The inhibitory effect of PGG and DOX on cell proliferation in TNBC cells, as assessed using the MTT assay. (**A**–**C**) Dose–response curves illustrating the cytotoxic effects of PGG on MCF-10A cells (**A**), DOX on MDA-MB231 cells (**B**), and PGG on MDA-MB231 cells (**C**) after 48 h of treatment. (**D**,**E**) Clonogenic survival analysis indicating the number of colonies formed by MDA-MB231 cells following treatment with varying concentrations of PGG (0, 2.5, 5, 10, 20, and 40 µM). Representative colony images are shown in (**D**), with quantification graphs in (**E**). The results are presented as the mean ± SD from three independent experiments. * *p* < 0.05 indicates a statistically significant difference compared to the control, while *** *p* < 0.001 denotes a highly significant difference from the control. PGG, pentagalloyl glucose; DOX, doxorubicin.

PGG, a natural compound, has minimal side effects, which is a significant advantage. Initially, the normal human breast epithelial cells (MCF-10A) were treated with PGG for 48 h. As shown in Figure 1, PGG had less cytotoxicity towards normal breast MCF-10A cells, with an IC_50_ exceeding 100 µM at 48 h of incubation time. Subsequently, we assessed the inhibition of cell growth by PGG and doxorubicin (DOX) in human TNBC cell lines. The MDA-MB231 cells were subjected to different concentrations of PGG and DOX for 48 h before cell viability analysis using the MTT assay. PGG exhibited a dose-dependent inhibition of the TNBC cell line growth, with IC_50_ values measured at 37 ± 7.05 µM. DOX also inhibited the TNBC cell growth in a dose-dependent manner, displaying IC_50_ values of 1.51 ± 0.15 µM. We then performed colony formation assays in order to evaluate the prolonged inhibitory effects of PGG. The MDA-MB231 cells were exposed to varying concentrations of PGG and allowed to proliferate for 14 days. The findings demonstrated a dose-dependent inhibition of colony formation by PGG, which was consistent with the outcomes of the cell viability assay.

### 2.2. PGG Suppresses Invasion and Migration Potential and Alters the Expression Levels of EMT-Associated Proteins in Triple-Negative Breast Cancer Cells

**Figure 2:**
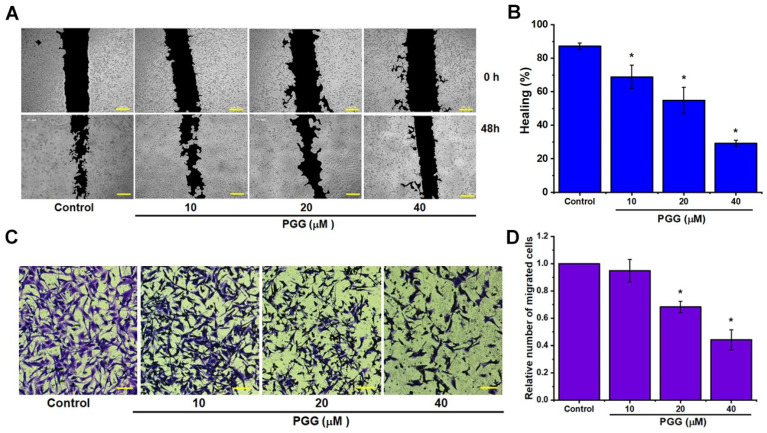
PGG inhibits the migration and invasion capabilities of TNBC cells. (**A**) Representative microscopic images from wound healing assays performed on MDA-MB231 cells treated with varying concentrations of PGG, captured at 0 and 48 h. (**B**) Quantification of wound closure percentages, demonstrating the impact of PGG treatment. (**C**) Transwell chamber images showing cell migration following treatment with various concentrations of PGG. (**D**) Quantification of migrated cells: migrating cells were counted in five high-power fields and averaged. The results are presented as the mean ± SD from three independent experiments. * *p* < 0.05 indicates a statistically significant difference compared to the control. Scale bar = 100 µm (**A**,**C**). PGG, pentagalloyl glucose.

As migration and invasion are pivotal mechanisms in tumor progression and metastasis, we investigated the impact of PGG on these processes in breast cancer cells. Using wound healing and Transwell migration assays, we evaluated the effect of PGG on the motility and migration abilities of MDA-MB231 cells, which are known for their high metastatic potential. Following a 48 h treatment with PGG, a significant reduction in migratory activity was observed, as evidenced by the decreased movement of cells into the scarred region compared to the control group, as shown in Figure 2. The untreated MDA-MB231 cells displayed substantial wound closure by the 24 h mark. In contrast, the wounds treated with PGG exhibited dose-dependent delays in healing. These findings were consistent with the results from the Transwell migration assay, where PGG treatment led to a dose-dependent decrease in the relative number of migrated cells, declining from 1 in the control group to approximately 0.95 at a PGG dose of 10 µM, 0.68 at a PGG dose of 20 µM, and around 0.44 at a PGG dose of 40 µM. Our results demonstrated the effectiveness of PGG in inhibiting migration in breast cancer cells.

### 2.3. PGG Enhances the Antitumor Effect of Doxorubicin in Triple-Negative Breast Cancer Cells

**Figure 3:**
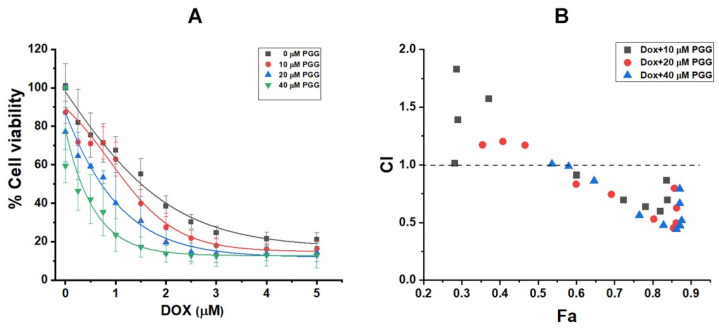
Impact of combined treatment with 10, 20, and 40 µM PGG and varying concentrations of DOX on the viability of MDA-MB231 cells after 48 h, as assessed using the MTT assay (**A**). Fa-CI plot analysis depicting the interaction between DOX and PGG in MDA-MB231 cells. The dashed line at CI = 1 signifies an additive effect, while CI values less than, equal to, or greater than 1 indicate synergy, additivity, or antagonism, respectively (**B**). The effect (Fa) represents the degree of fractional inhibition associated with each combination index. PGG, pentagalloyl glucose; DOX, doxorubicin.

Given the challenges involved in treating triple-negative breast cancers with conventional chemotherapy, our study set out to investigate a promising alternative. We aimed to assess whether the combination of PGG with DOX could significantly enhance therapeutic outcomes. To this end, we co-treated MDA-MB231 cells with increasing concentrations of DOX (0–5 µM) and fixed concentrations of PGG at 10, 20, and 40 µM for 48 h. The subsequent measurement of cell viabilities using the MTT assay provided intriguing insights into the potential of this combination. The results, as shown in Figure 3, revealed that DOX treatment alone led to dose-dependent growth inhibition in MDA-MB231 cells, with an IC_50_ value of 1.54 ± 0.12 μM. However, co-treatment with DOX and PGG at concentrations of 10, 20, and 40 μM significantly enhanced growth inhibition, as evidenced by the markedly decreased IC_50_ values of 1.22 ± 0.18, 0.89 ± 0.24, and 0.312 ± 0.16 μM, respectively. The Chou–Talalay combination index (CI) for DOX and PGG was conducted using CompuSyn software version 1.0, indicating effects ranging from antagonistic to synergistic depending on the concentration of PGG. As depicted in Figure 3, CI values were observed to fall below, equal to, or above the additive line (CI = 1). The Fa-CI plot revealed synergism, with CI values below 1, for most combinations of 40 µM PGG and DOX across nearly all effect levels. Conversely, combinations with PGG at 10 and 20 µM showed CI values greater than 1 at certain concentrations, indicating antagonistic interactions under those conditions. At the 50% drug effect level (ED50), the CI values were calculated as 1.09, 0.82, and 0.52 for combinations with PGG at 10, 20, and 40 µM, respectively. A CI value of less than one indicates a synergistic effect, while a value greater than one indicates an antagonistic effect. Notably, synergism was observed in the MDA-MB231 cells with PGG concentrations of 20 and 40 µM. Moreover, our study demonstrated a significant reduction in the dosage of DOX, which is a potent chemotherapeutic drug, due to the synergistic behavior of PGG. The dose reduction index (DRI) revealed that the IC_50_ of DOX decreased by 1.26-, 1.73-, and 6.42-fold for PGG concentrations of 10, 20, and 40 µM, respectively. These findings underscore the synergistic cytotoxicity of DOX and PGG in triple-negative breast cancer MDA-MB231 cells and also offer a potential strategy to mitigate the side effects of high-dose chemotherapy.

### 2.4. Combination Treatment of PGG and Doxorubicin Enhances Apoptosis in Triple-Negative Breast Cancer Cells

**Figure 4:**
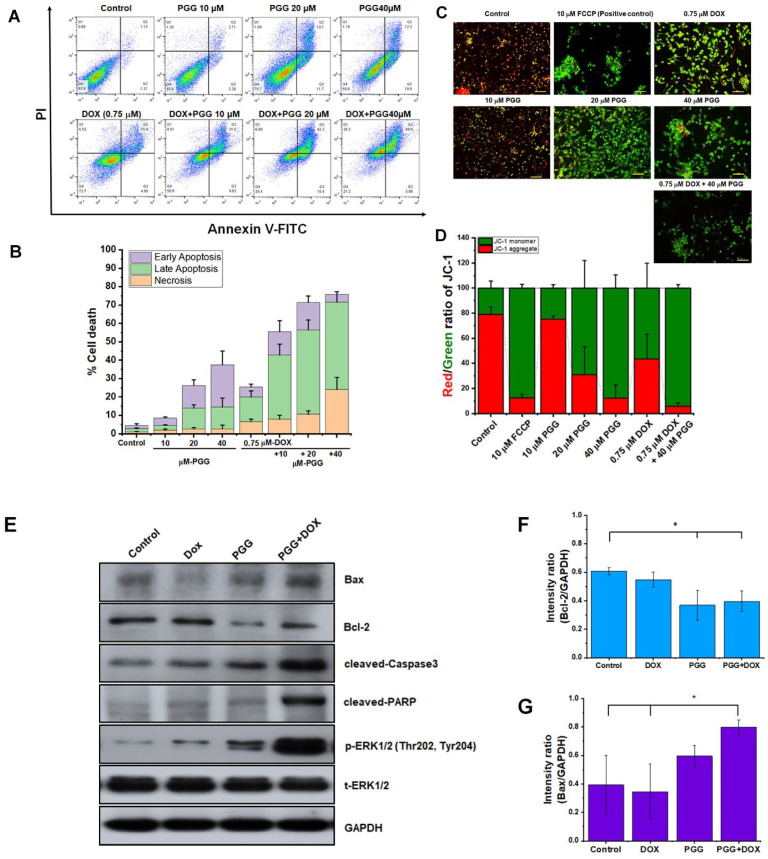

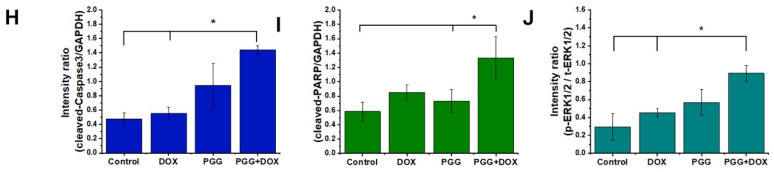
The impact on apoptosis of PGG and DOX as monotherapies or in combination in TNBC cells. (**A**) Representative dot plots show the apoptotic response of MDA-MB231 cells to the indicated treatments. (**B**) Quantitative data represent the percentage of total cell death, as determined using flow cytometry. (**C**,**D**) The effects of PGG on mitochondrial membrane potential in MDA-MB231 cells were assessed using JC-1 staining: (**C**) representative images display JC-1 fluorescence across different treatment groups after 24 h, with FCCP as the positive control. Monomeric JC-1 exhibits green fluorescence, while aggregated JC-1 emits red fluorescence; (**D**) quantification of the red/green fluorescence ratio shown in a histogram. Data are presented as the mean ± SD of three independent experiments. (**E**) Western blot analysis of apoptotic markers, including Bax, Bcl-2, caspase-3, PARP, p-ERK, and t-ERK. The uncropped Western blot images are provided in Figure S1. (**F**–**J**) The relative protein density values were quantified, with expression levels normalized to GAPDH as the loading control. The results are presented as the mean ± SD from three independent experiments. * *p* < 0.05 indicates a statistically significant difference compared to the control. Scale bar = 100 µm (**C**). PGG, pentagalloyl glucose; DOX, doxorubicin; PARP, poly ADP ribose polymerase; ERK, extracellular signal-regulated kinase.

Next, we sought to determine whether the synergistic cytotoxic effect of PGG and DOX was associated with apoptosis. The staining results of annexin V/PI, a widely used method to detect apoptotic cells, indicated higher apoptotic rates in the MDA-MB231 cells co-treated with PGG and DOX compared to the groups receiving single treatments, as shown in Figure 4.

Our study involved evaluation of the mitochondrial membrane potential (ΔΨ_m_), which is a crucial measure of apoptosis. The loss of ΔΨ_m_ is an early indicator of apoptosis, indicating compromised mitochondrial membrane integrity. To investigate whether PGG triggers apoptosis through the mitochondria-mediated pathway, we used JC-1 staining. JC-1 is a positively charged dye that specifically enters mitochondria and functions as a probe that emits two different wavelengths of light. JC-1 exists as a monomer and exhibits green fluorescence at low ΔΨ_m_; at high ΔΨ_m_, it forms aggregates and emits red fluorescence. The result shows that in the control group, JC-1 produced red fluorescence. After 24 h of exposure to PGG, DOX, or a combination of both, the MDA-MB231 cells showed a predominance of green fluorescence from JC-1, with minimal red fluorescence. This transition from red to green fluorescence indicates that PGG and DOX, alone or together, lead to the dispersion of MMP. We further analyzed ΔΨ_m_ using flow cytometry. The analysis showed a higher increase in green fluorescent intensity, which is indicative of monomeric JC-1, following combination treatment with PGG and DOX compared to the cells treated with PGG or DOX alone. The untreated cells exhibited 81.53% red and 18.47% green, suggesting higher ΔΨ_m_ values. Additionally, the findings revealed an amplified apoptotic effect with the combined treatment, as shown by reduced Bcl-2 expression and elevated levels of Bax, cleaved caspase-3, and cleaved PARP. These results underscore that PGG and DOX, in combination, predominantly induce apoptotic cell death in triple-negative breast cancer cells through a mitochondrial-dependent mechanism.

In addition, extracellular signal-regulated kinase (ERK), belonging to the MAPK (mitogen-activated protein kinase) family, governs cellular proliferation and viability. Although conventionally linked to cell growth, ERK can promote cell death under specific contexts. Recent studies have indicated that chemopreventive agents can induce cancer cell death under particular conditions by activating ERK pathways [32]. This study investigated the involvement of ERK signaling pathways in facilitating apoptotic cell death induced by the combined treatment of PGG and DOX. The findings revealed that treatment with PGG or DOX individually led to an increase in phosphorylated ERK protein levels. Interestingly, the combination treatment of these two compounds led to even greater phosphorylation of ERK. These results indicate that the enhanced activation of ERK signaling pathways promotes apoptotic cell death in triple-negative breast cancer cells treated with the combination of PGG and DOX. Collectively, these results suggest that the combination of PGG and DOX works synergistically to promote apoptosis in triple-negative breast cancer cells.

### 2.5. PGG and Doxorubicin Combination Enhances the Anti-Migration Effect of DOX in Triple-Negative Breast Cancer Cells

**Figure 5:**
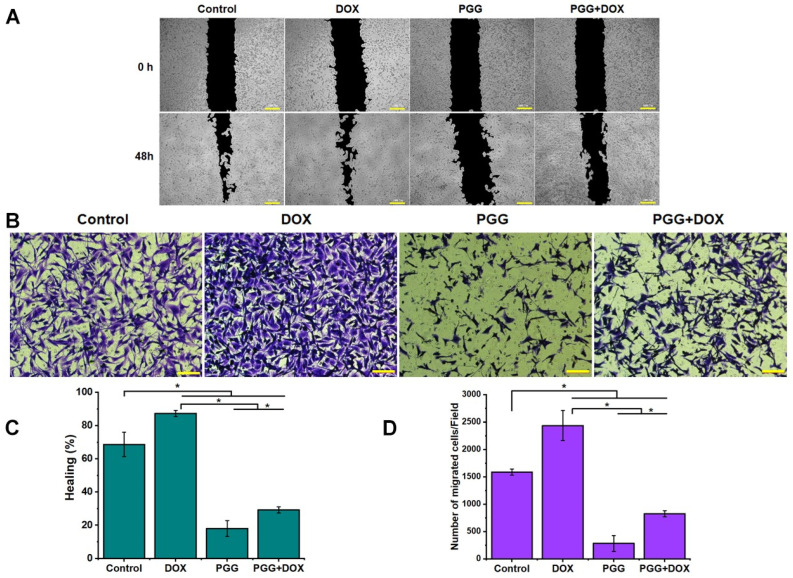
The impact of PGG combined with DOX on the migratory behavior of TNBC cells. (**A**) Representative microscopic images from wound healing assays and (**C**) quantification of wound closure percentages illustrating the effects of treatments with PGG (40 µM), DOX (0.75 µM), and their combination. (**B**) Transwell chamber images showing cell migration following treatment with PGG (40 µM), DOX (0.75 µM), or a combination of both. (**D**) Quantitative analysis of the number of migrating cells. The results are presented as the mean ± SD from three independent experiments. * *p* < 0.05 indicates a statistically significant difference compared to the control and DOX treatment alone. Scale bar = 100 µm (**A**,**B**). PGG, pentagalloyl glucose; DOX, doxorubicin.

The effect of combining PGG with DOX on MDA-MB231 cell migration was evaluated through scratch wound healing and Transwell migration assays. As shown in Figure 5, the scratch wound healing assay demonstrated that cells in the control group and those treated with a sub-lethal dose of DOX (0.75 µM) migrated rapidly, resulting in swift wound closure, indicating that this dose of DOX did not impede the migration ability of MDA-MB231 cells. Conversely, the treatment with 40 µM PGG effectively suppressed cell migration towards the wound, resulting in a significant gap in the wound area. Furthermore, combining PGG with a sub-lethal dose of DOX augmented the inhibitory effects on MDA-MB231 cell migration compared to either DOX treatment alone or the control group. These findings underscore the enhanced migratory inhibition achieved with the combined PGG and DOX treatment. Additionally, the results from the Transwell migration assay mirrored these observations, with the combination of PGG and the sub-lethal dose of DOX significantly reducing the invasion of MDA-MB231 cells compared to the control or DOX-only groups. Notably, the combination treatment exhibited a substantially stronger inhibition of migration compared to DOX alone, which can stimulate the migration of TNBC cells at low doses. This provides compelling evidence of the combination’s enhanced ability to impede the migration of these highly metastatic cells while improving the efficacy of DOX in controlling their movement.

### 2.6. PGG-Mediated Reversal of the EMT Process Plays a Crucial Role in the Anti-Migration Effect Induced by Combination Treatment of PGG and DOX

**Figure 6:**
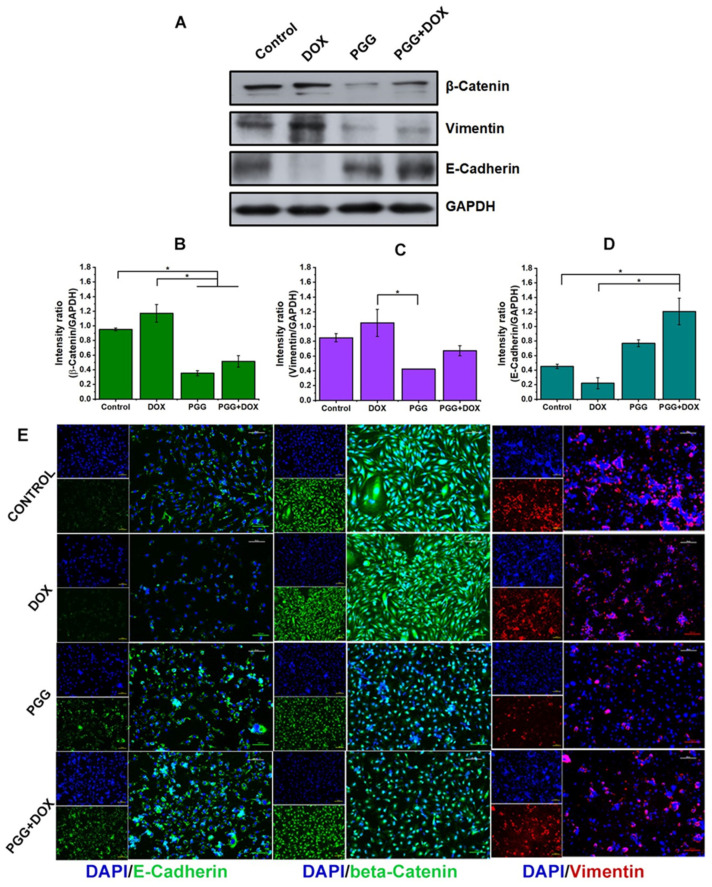
Reversal of EMT and suppression of EMT marker expression by PGG in TNBC cell lines. MDA-MB231 cells were treated for 48 h, as indicated, and the expression of EMT markers was analyzed using Western blot. (**A**) Representative Western blot images showing the levels of β-catenin, vimentin, E-cadherin, and GAPDH. The uncropped Western blot images are provided in Figure S2. (**B**–**D**) Quantification of band intensities from the Western blot analysis. Relative protein levels were quantified and normalized to GAPDH as the loading control. The results are presented as the mean ± SD from three independent experiments. * *p* < 0.05 indicates a statistically significant difference compared to the control and DOX-only treatment. (**E**) Immunofluorescence staining of β-catenin (green), E-cadherin (green), and vimentin (red) in MDA-MB231 cells, with nuclei counterstained using DAPI (blue). Scale bar = 100 µm.

Our inquiry aimed to examine the possible correlation between the inhibitory impact of PGG on migration and the reduction or reversal of EMT. We employed Western blot and immunofluorescent assays to investigate the expression levels of EMT-related proteins in MDA-MB231 cells treated with DOX, PGG, or a combination of both for 48 h. The results, shown in Figure 6, revealed that DOX treatment increased the expression of the mesenchymal cell markers β-catenin and vimentin but decreased the expression of E-cadherin, an adhesion molecule for epithelial cells, in the MDA-MB231 cells. In contrast, the PGG treatment, whether alone or combined with DOX, resulted in decreased vimentin and β-catenin expression, while it increased the E-cadherin expression prominently. Notably, when DOX was present, PGG significantly counteracted the DOX-induced upregulation of β-catenin and vimentin. Furthermore, compared to either treatment alone, the combined treatment with DOX and PGG led to a significant downregulation of vimentin and β-catenin expression, while it increased E-cadherin expression, which was consistent with the observed suppression of cell migration. The immunofluorescent assays confirmed similar alterations in the E-cadherin, β-catenin, and vimentin cellular expression patterns. These findings have significant implications for cancer research, suggesting that PGG has the potential to counteract DOX-induced EMT; this is achieved by downregulating vimentin and β-catenin expression while upregulating E-cadherin expression, thereby providing a novel approach to the attenuation or reversal of EMT.

### 2.7. Abrogation of STAT3 Is Integral to PGG-Mediated Inhibition of EMT, Invasion, and Migration of Triple-Negative Breast Cancer Cells

**Figure 7:**
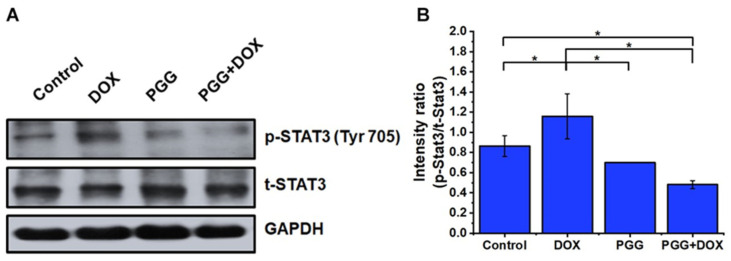
The effects of PGG and DOX, either alone or in combination, on STAT3 signaling proteins in TNBC cells. (**A**) Representative Western blot images showing the expression levels of phosphorylated STAT3 (p-STAT3), total STAT3 (t-STAT3), and GAPDH after treatment with PGG (40 µM), DOX (0.75 µM), or their combination for 48 h in MDA-MB231 cells. The uncropped Western blot images are provided in Figure S3. (**B**) Bar graph depicting the fold change in protein expression. The relative protein densities were quantified and normalized to the GAPDH loading control. The results are presented as the mean ± SD from three independent experiments. * *p* < 0.05 indicates a statistically significant difference compared to the control and DOX treatment alone. PGG, pentagalloyl glucose; DOX, doxorubicin; STAT3, signal transducer and activator of transcription 3; p, phosphorylated; t, total; GAPDH, glyceraldehyde 3-phosphate dehydrogenase.

The sustained stimulation of signal transducer and activator of transcription 3 (STAT3) has been linked to several cancers, including breast cancer. STAT3 is a key regulator promoting the proliferation of breast cancer stem cells [33,34]. Its activation regulates the expression of numerous gene products involved in anti-apoptosis, invasion, and angiogenesis. Furthermore, evidence suggests that the activation of STAT3 is associated with the EMT process and cancer progression [35,36]. Our objective was to investigate whether the inhibitory effect of PGG on MDA-MB231 cells was associated with the modulation of STAT3 phosphorylation and activation. The MDA-MB231 cells were treated with DOX, PGG, or a combination of both for 48 h. Western blot analysis was used to evaluate the expression levels of phosphorylated STAT3, as shown in Figure 7. The treatment with DOX alone led to increased levels of phosphorylated STAT3 at Tyr-705, whereas the treatment with PGG alone markedly suppressed STAT3 phosphorylation, with no discernible changes in total STAT3 levels observed. The combination of PGG and DOX exerted an additive effect, further reducing STAT3 phosphorylation. Notably, the combination treatment with PGG dramatically inhibited DOX-induced active STAT3. Therefore, we hypothesize that PGG can inhibit the EMT process and enhance DOX sensitivity by abolishing STAT3 activation in MDA-MB231 cells.

## 3. Discussion

Despite its potent anticancer properties, doxorubicin (DOX) has limited clinical application due to its toxicity to normal tissues, such as the heart and liver, and the development of multidrug resistance in cancer cells. There is a growing interest in the combination of chemotherapeutic medications with naturally occurring phytochemicals. The therapeutic outcomes of cancer patients have been significantly improved by the use of numerous compounds derived from natural products when combined with chemotherapeutic drugs. These combinations can reduce side effects, surmount drug resistance, and induce synergistic effects [37,38].

PGG, a gallotannin isolated from *Bouea macrophylla* plants, is known for its strong antitumor activities in various cancers, including lung, prostate, breast, and liver cancers [25]. Previous studies have shown that PGG modulates critical cellular processes, such as apoptosis, angiogenesis, metastasis, and signaling pathways [26,27,28,39]. Importantly, these studies have also highlighted the promising safety profile of PGG in animal models, providing reassurance about its potential use in cancer treatment [40]. Previous research has indicated that PGG and the conventional chemotherapeutic drug 5-FU exhibit a synergistic effect in the treatment of hepatocellular carcinoma. This combination promoted the activation of caspase-9 and caspase-3 and increased the Bax/Bcl-2 ratio, thereby inducing apoptosis; it also demonstrated synergistic effects on the aggressive phenotypes of HepG2 cells. PGG’s ability to downregulate multidrug resistance protein 1 (MDR1) and low-density lipoprotein receptor-related protein 1 (LRP1) is particularly noteworthy, as it suggests its potential to combat drug resistance [41]. This in vitro study demonstrated that the combined treatment of PGG and DOX effectively reduced the growth and metastatic potential of MDA-MB231 triple-negative breast cancer (TNBC) cells. The combination decreased the required DOX dose by 1.26-, 1.73-, and 6.42-fold for PGG at 10, 20, and 40 µM, respectively, while maintaining equivalent growth inhibition rates. Preliminary tests on MCF-7, a DOX-sensitive breast cancer cell line, showed no enhanced effect with the combination therapy, likely due to MCF-7’s inherent sensitivity to DOX, minimizing the need for additional synergistic effects. These results underscore the specificity and therapeutic relevance of the PGG + DOX combination in overcoming DOX resistance, a major challenge in TNBC treatment, and affirm its potential as an effective strategy for aggressive breast cancer subtypes. Importantly, the highest concentration of PGG (40 µM) in the combination therapy did not exhibit cytotoxicity in normal breast epithelial MCF-10A cells, emphasizing PGG’s selective action against cancer cells. Therefore, this concentration was used in subsequent experiments. Our results indicate that PGG, when combined with DOX, enhances anticancer effects at lower concentrations, reducing the adverse effects typically associated with higher doses of DOX. This is the first study to demonstrate the synergistic effectiveness of PGG combined with DOX against TNBC. Our findings suggest that the combination treatment with PGG and DOX could pave the way for a novel, more effective, and less toxic chemotherapeutic strategy for breast cancer. Our findings demonstrated that both PGG and DOX suppressed proliferation and induced apoptosis in TNBC cell lines in a dose-responsive manner. Furthermore, this combination treatment significantly amplified the apoptotic effect of DOX, underscoring its potential as a transformative approach in breast cancer therapy.

DOX induces apoptosis through two key mechanisms: the extrinsic pathway, involving the activation of caspase-8, and the intrinsic or mitochondrial pathway, driven by mitochondrial release of pro-apoptotic factors and subsequent activation of caspase-9, with the intrinsic pathway being the predominant route [42]. The mitochondrial pathway entails the permeabilization of the outer mitochondrial membrane, which releases pro-apoptotic factors such as cytochrome c into the cytosol, leading to the formation of the apoptosome and the activation of effector caspases [43]. In our study, we investigated the potential synergistic interaction between PGG and DOX in TNBC cells. We found that PGG significantly enhanced DOX-induced mitochondrial membrane potential loss, promoting mitochondrial breakdown and the subsequent cytochrome c release and caspase activation. This suggests that PGG sensitizes TNBC cells to DOX-induced apoptosis primarily through the mitochondrial pathway. Compared to treatment with DOX alone, the combined treatment with PGG promoted PARP cleavage and stimulated effector caspase-3 activation, leading to the initiation of apoptotic pathways. Interestingly, we also noted the upregulation of extracellular signal-regulated kinase (ERK) in the combination treatment, which is known to play a dual role in cancer biology by either promoting survival or inducing apoptosis, depending on the context [44]. In the case of our combination treatment, the sustained activation of ERK appears to enhance apoptosis rather than promote cell survival. This pro-apoptotic role of ERK can be attributed to its ability to phosphorylate and stabilize p53, thereby enhancing its transcriptional activity on pro-apoptotic genes such as Bax and Bim [45]. Additionally, ERK may inhibit anti-apoptotic signals, tipping the balance toward cell death. This upregulation of ERK in the combination treatment further amplifies the apoptotic signaling, thereby enhancing the overall efficacy of the treatment and reducing the required dose of DOX, potentially mitigating its associated side effects.

Migration and invasion are critical steps in cancer metastasis. Recent studies have suggested that chemotherapeutic agents may initiate or accelerate the development of metastases [46]. Our findings revealed that TNBC cells acquired EMT traits following DOX treatment, leading to the upregulation of proteins associated with cell motility, which ultimately enhanced the migratory ability of the cancer cells (Figure 5 and Figure 6). Our findings align with those reported in prior studies showing that chemotherapeutic agents, such as doxorubicin and paclitaxel, can induce drug resistance and EMT in treated cancers [47,48]. EMT refers to the transformation of epithelial cells into mesenchymal cells with enhanced abilities to migrate, invade, and resist therapeutics. Interestingly, we observed that PGG independently suppressed the motility of TNBC cells, which are known for their high metastatic potential, without causing toxic effects. Even more inspiring, the combination therapy of DOX and PGG was more potent in inhibiting cell migration than DOX and PGG monotherapies. This potent effect is likely due to PGG’s ability to suppress the DOX-induced EMT process, thereby inhibiting cell migration. Our results also showed that PGG could reverse doxorubicin-acquired EMT properties, downregulating β-catenin and vimentin, upregulating E-cadherin, and maintaining cell−cell contact in TNBCs. The suppression of the EMT process by PGG has been previously documented. Studies have reported that PGG derived from Maprang seed extract (MPSE) inhibits the radiation-induced EMT process and further enhances its radiation-sensitizing properties when used in combination with radiation [29]. These results underscore the potential of PGG to enhance the efficacy of cancer treatment when used in combination with conventional therapy.

The STAT3 pathway, a crucial regulator of various cellular processes, including proliferation, survival, and differentiation, is often aberrantly activated in many cancers, including TNBC [33,34]. Our findings demonstrated that activating the STAT3 pathway promotes the EMT process in TNBC cells. This activation leads to the transcription of several target genes that drive EMT, including transcription factors such as β-catenin. These factors suppress epithelial characteristics and promote mesenchymal traits, thereby enhancing the invasive potential of TNBC cells. In contrast, PGG treatment plays a crucial role in restoring E-cadherin expression and reducing the levels of mesenchymal markers, thereby inhibiting the migratory and invasive capabilities of TNBC cells. In our study, we noted that PGG treatment reduced the phosphorylation of STAT3 in MDA-MB231 cells exposed to PGG. Furthermore, our study revealed that combining PGG with DOX potentiated the anticancer effects of DOX and significantly mitigated DOX-induced EMT. DOX treatment alone was found to promote EMT through STAT3 activation, contributing to increased metastasis and chemoresistance. However, co-treatment with PGG, by suppressing STAT3 activation and reversing the EMT process induced by DOX, highlighted the potential of PGG to counteract the adverse effects of chemotherapy and enhance its efficacy, offering an encouraging prospect for TNBC treatment.

This study underscores the potential of PGG as a promising therapeutic agent in combination with DOX for triple-negative breast cancer (TNBC). However, several challenges must be overcome to ensure clinical translation. PGG, a naturally occurring compound, is abundant in sources such as *Bouea macrophylla* seeds, a biowaste product with high extraction yields (52.88 g/kg), second only to gallnuts (58.40 g/kg) [25]. Employing modern extraction and purification techniques could enable large-scale production in an environmentally and economically sustainable manner, ensuring scalability for clinical and commercial applications. As a polyphenolic compound with diverse biological activities, PGG may interact with unintended molecular targets, warranting careful evaluation of potential off-target effects. While in vitro data suggest minimal toxicity in normal breast epithelial cells, previous studies also report low toxicity for PGG at high doses. For instance, oral doses of 100–200 mg/kg/day were shown to be safe in mice, with no observable toxicity or organ weight changes [40]. Furthermore, intraperitoneal injections of PGG (10–15 mg/kg) significantly inhibited liver and lung metastases in colon cancer metastatic mouse models without causing toxicity to normal tissues [26]. However, additional in vivo studies are essential to confirm its safety in sensitive organs, such as the liver and kidneys, particularly when used in combination with chemotherapy. Future research should prioritize validating PGG’s efficacy and safety through comprehensive in vivo studies. Moreover, critical limitations of this study must be addressed, including evaluating the therapeutic efficacy of the combination treatment in in vivo models and using STAT3 inhibitors to confirm whether STAT3 signaling is the primary target of PGG + DOX therapy. Addressing these challenges will enhance PGG’s translational potential as a natural therapeutic agent.

## 4. Materials and Methods

### 4.1. Preparation of PGG (Penta-O-galloyl-β-D-glucose)

PGG was isolated from the crude seed extract of *Bouea macrophylla* Griffith following a previously established protocol [49]. Firstly, 1 g of the crude extract was dissolved in 1 L of deionized water to create the *Bouea macrophylla* seed extract (MPSE) at a concentration of 1 g/L. The solution was frozen at −20 °C for 3 h and then thawed at 4 °C over the course of 3 h at room temperature. Subsequently, the precipitate was carefully washed with 50 mL of chilled water and centrifuged at 2500× *g* for 20 min (Andreas Hettich GmbH & Co. KG, Tuttlingen, Germany). This washing and centrifugation process was repeated five times to ensure thorough purification of the precipitate. The resulting PGG pellets were then lyophilized into a powdered form and stored in a desiccator at room temperature for future applications. The quantification of PGG was performed using a previously validated method employing a Shimadzu LC-20AD prominence liquid chromatograph system with an SPD-M20A prominence diode array detector (Shimadzu, Nakagyo-Ku, Kyoto, Japan). This system ensured the accuracy of our results, confirming the purity of PGG at 97% and providing a high level of confidence in our findings. To prepare a stock solution of PGG at a concentration of 1 mg/mL, the PGG compound was dissolved in 1 mL of deionized water and sonicated for 30 min. To eliminate any potential pathogen contaminants, the solution was sterilized by passing it through a 0.22 µm membrane filter. The sterilized solution was then divided into aliquots and stored at −20 °C for subsequent experimental use. Doxorubicin hydrochloride was sourced from Sigma Aldrich (St. Louis, MO, USA). To prepare the stock solutions, 2 mg of doxorubicin was dissolved in 1 mL of deionized water to yield a final 2 mg/mL concentration. The solution was mixed completely to guarantee the complete dissolution of the doxorubicin.

### 4.2. Cell Line and Cell Culture

The human triple-negative breast cancer cell line MDA-MB231 (ATCC^®^ HTB-26™) was sourced from the American Type Culture Collection (Manassas, Virginia, USA). These cells were carefully maintained in Roswell Park Memorial Institute medium (RPMI-1640) supplemented with 2 mM L-glutamine (Gibco, Carlsbad, CA, USA), 10% fetal bovine serum (FBS; Gibco, Carlsbad, CA, USA), and 1% penicillin–streptomycin (10,000 U/mL; Gibco, Carlsbad, CA, USA). The cell cultures were incubated at 37 °C in a humidified environment with 5% CO_2_.

### 4.3. Cell Viability Assay

MDA-MB231 cells at a density of 5 × 10^3^ cells per well were used to seed into 96-well plates, and the plates were left overnight to facilitate cell attachment and growth. The cells were treated with various concentrations of doxorubicin (0–5 µM), PGG (0–110 µM), and a combination of both compounds for 48 h. The 3-(4,5-dimethylthiazol-2-yl)-2,5-diphenyltetrazolium bromide (MTT) assay was utilized to assess cell viability. The formazan solution absorbance was quantified at a wavelength of 560 nm using a microplate reader (BioTek™ Eon™ microplate reader, Winooski, VT, USA). Relative cell viability was determined by dividing the absorbance of treated cells by that of the control group. The half-maximal inhibitory concentration (IC_50_) was calculated using data from three independent experiments and analyzed with OriginPro version 2023 software (Origin Lab, Northampton, MA, USA). The interaction between the two compounds was assessed by calculating the combination index (CI) through the Chou–Talalay method using the CompuSyn software Version 1.0 package (Biosoft, Ferguson, MO, USA). A Fa-CI plot (fraction affected–combination index) was generated to characterize the interaction. A CI value of less than one suggested a meaningful synergistic interaction between the compounds.

### 4.4. Colony Formation Assay

MDA-MB231 cells were seeded as single cells into 6-well plates and allowed to adhere overnight. Afterward, the cells were exposed to PGG, doxorubicin, or a combination of the two drugs for 48 h. Following the treatment, the cells were cultured for an additional 14 days to facilitate colony formation. The resulting colonies were fixed using a fixation solution composed of three parts methanol and one part acetic acid (3:1 ratio) at room temperature for 30 min. Afterward, the colonies were stained with 0.5% crystal violet solution. Colonies containing more than 50 cells were captured and quantified using an inverted microscope (ECLIPSE Ts2, Nikon, Tokyo, Japan).

### 4.5. Apoptosis Assay by Annexin V-FITC/PI Double Staining

Cells were seeded into 6-well plates and left to adhere for 24 h prior to treatment with different concentrations of PGG, doxorubicin, or a combination of the two drugs for 48 h. Following treatment, the cells were harvested by detaching them with a trypsin–EDTA solution and subsequently rinsed with 1× binding buffer. The collected cells were then subjected to dual staining with annexin V-FITC and propidium iodide (PI) for 20 min at room temperature, protected from light, following the manufacturer’s instructions. Immediately after staining, the cells were examined via flow cytometry using CytoFLEX (Beckman Coulter, Brea, CA, USA). The flow cytometric data were processed and analyzed using FlowJoTM software, version 10 (Becton, Dickinson & Company, Franklin Lake, NJ, USA).

### 4.6. Mitochondrial Membrane Potential

The qualitative and quantitative alterations in mitochondrial membrane potential (MMP) were assessed using the JC-1 staining kit from Sigma-Aldrich (St. Louis, MO, USA). In healthy cells, JC-1 accumulates within the mitochondria, emitting intense red fluorescence with excitation at 560 nm and emission at 595 nm. Conversely, JC-1 remains monomeric in apoptotic cells, emitting a strong green fluorescence with excitation and emission at 485 and 535 nm, respectively. MDA-MB231 cells were plated in 6-well plates at a density of 2 × 10^5^ cells per well and incubated for 24 h to allow adherence. Following this, the cells were exposed to different concentrations of PGG, doxorubicin, or a combination of both for a duration of 24 h. Post-treatment, the cells were harvested and incubated with 3 µg/mL JC-1 dye in an incubation buffer for 20 min at 37 °C. Following this careful incubation, the cells were washed, and images were acquired using a fluorescence microscope (ECLIPSE Ts2, Nikon, Tokyo, Japan). The stained cells were meticulously analyzed via quantitative analysis using a flow cytometer, CytoFLEX (Beckman Coulter, Brea, CA, USA). The flow cytometer detected red fluorescence from the JC-1 aggregates, indicating higher membrane potentials, and green fluorescence from the JC-1 monomers, indicating lower membrane potentials. The mitochondrial membrane potential, a key parameter in our study, was determined by calculating the red-to-green fluorescence intensity ratio.

### 4.7. Wound Healing Assay

A wound healing assay was performed to evaluate the migratory capacity of MDA-MB231 cells. Cells in the logarithmic growth phase were seeded into 6-well plates at a density of 5 × 10^5^ cells per well. The next day, once the cells achieved nearly full confluence, a sterile pipette tip was used to carefully create a defined, cell-free gap in the monolayer. The detached cells were meticulously removed by washing the wells twice with PBS. The remaining cells were then incubated with PGG, doxorubicin, or a combination of both drugs in serum-free RPMI-1640 medium for 24 h at 37 °C. Images of the wound were captured at the time of scratching (0 h) and after 48 h using an inverted microscope at 10× magnification, revealing significant insights into the effects of these compounds on wound healing.

The migration distance was calculated using the following formula:

Migration distance = (scratch distance at 0 h) − (scratch distance at 48 h) (1)

### 4.8. Transwell Migration Assay

To evaluate the invasive capabilities of the MDA-MB231 cells, a Transwell migration assay was conducted using a 24-well plate setup with the Transwell insert. The cells were cultured in serum-free RPMI-1640 medium for 24 h at 37 °C; this condition was chosen to minimize the influence of external factors, such as growth factors, in the serum. The upper chamber was then filled with 100 µL of cell suspension containing 1 × 10^4^ cells per chamber in serum-free RPMI-1640 medium with PGG, doxorubicin, or a combination of both drugs. The lower chamber was filled with 700 µL of RPMI-1640 medium supplemented with 20% FBS to serve as a chemoattractant. After incubating for 24 h at 37 °C, cells that had not migrated and remained on the upper side of the membrane were gently removed using a cotton swab. Cells that traversed to the underside of the insert membrane were immobilized using 4% paraformaldehyde for 15 min at room temperature. After fixation, the cells were stained with 0.1% crystal violet for 30 min. Photographs of the stained invasive cells were taken, and their numbers were carefully quantified in five randomly selected fields of view using an inverted microscope at 10× magnification.

### 4.9. Western Blot Analysis

The MDA-MB231 cells were treated with PGG, doxorubicin, or a combination of both drugs for 48 h. After treatment, the cells were treated with CelLytic M (Sigma-Aldrich, St. Louis, MO, USA) lysis buffer on ice for 30 min supplemented with a 1% protease inhibitor cocktail to enable protein extraction. We then used the Bradford assay (Sigma-Aldrich, St. Louis, MO, USA) to quantify the protein quantities in the cell lysates. A 10% SDS-PAGE gel was loaded with 20 µg of the total protein from each sample to separate it for protein analysis. Following electrophoresis, the proteins from the gel were transferred to PVDF membranes (Millipore, St. Louis, MO, USA). The membranes were then blocked to prevent non-specific binding using 5% nonfat dry milk dissolved in Tris-buffered saline with Tween (TBS-T) for 1 h. The primary antibodies targeting total STAT3, phosphorylated STAT3, Bax, cleaved-caspase 3, cleaved-PARP, Bcl-2, vimentin, E-cadherin, β-catenin, and GAPDH were incubated overnight at 4 °C on blocked membranes. Following the primary antibody incubation, the membranes were extensively washed with TBS-T. Next, the membranes were incubated with horseradish peroxidase-conjugated secondary antibodies (diluted 1:10,000) for 1 h at room temperature. The protein bands were visualized using an enhanced chemiluminescence (ECL) detection system (Millipore, St. Louis, MO, USA), and the emitted signals were captured on X-ray film. The films were subsequently scanned, and the intensity of the bands was quantified using ImageMaster 2D Platinum software, version 5.0 (GE Healthcare Amersham Bioscience, Chicago, IL, USA). The expression levels of the target proteins were normalized to GAPDH, which served as the internal loading control to ensure accurate comparative analysis.

### 4.10. Immunofluorescence

The treatment of the MDA-MB231 cells with PGG, doxorubicin, or a combination of both drugs was conducted over a 48 h period. After the treatment, the cells were treated with 4% paraformaldehyde (PFA) for 20 min at room temperature to immobilize them and subsequently rinsed three times with PBS. The cells were then permeabilized with 0.2% Triton X-100 in PBS for 10 min and washed three times with PBS. To prevent non-specific binding, the cells were treated with 5% BSA in PBS for 1 h at room temperature. After blocking, they were incubated overnight at 4 °C with primary antibodies targeting β-catenin (1:200), E-cadherin (1:50), and vimentin (1:50). This was followed by thorough washing and incubation with secondary antibodies at a 1:200 dilution (Millipore, St. Louis, MO, USA) for 1 h at room temperature to ensure the accuracy of the results. The cells were then stained with a 1 µg/mL 4′,6-diamidino-2-phenylindole (DAPI) solution for nuclear staining. The results were visualized and captured using a fluorescence microscope (ECLIPSE Ts2, Nikon, Tokyo, Japan).

### 4.11. Statistical Analysis

Data from at least three separate experiments were expressed as the mean ± standard deviation (SD). Statistical differences among mean values were evaluated using one-way ANOVA, followed by post hoc analysis performed with the 2023 edition of OriginPro software (Origin Lab, Northampton, MA, USA). A *p*-value below 0.05 was regarded as statistically significant.

## 5. Conclusions

In conclusion, doxorubicin (DOX) is a potent anticancer drug with significant limitations due to its toxicity to normal tissues and the development of multidrug resistance in cancer cells. Combining DOX with naturally occurring phytochemicals, such as pentagalloyl glucose (PGG), has shown promising potential in overcoming these challenges. PGG, a gallotannin extracted from *Bouea macrophylla* plants, improves the treatment efficacy of DOX in managing triple-negative breast cancer (TNBC) by promoting apoptosis and reducing metastasis. Our study demonstrates that the combination of PGG and DOX effectively inhibits TNBC cell proliferation and migration while reducing the required dose of DOX, thus minimizing its adverse effects. PGG potentiates DOX-induced apoptosis primarily through the mitochondrial pathway and suppresses the DOX-induced epithelial–mesenchymal transition (EMT) by inhibiting the STAT3 pathway. The overall results indicate that PGG can boost the effectiveness of standard chemotherapy, offering a novel and less toxic chemotherapeutic strategy for TNBC treatment. Future research should focus on validating the therapeutic efficacy of this combination treatment in in vivo models. Addressing these challenges will further enhance PGG’s potential as a translational natural therapeutic agent.

## Data Availability

The data presented in this study are available on request from the corresponding author.

## Supplementary Materials

The following supporting information can be downloaded at www.mdpi.com/article/10.3390/ph17121729/s1, Figure S1: The uncropped Western blot images corresponding to Figure 4E showing all the bands. Red boxes indicate the samples of interest. Figure S2. The uncropped Western blot images corresponding to Figure 6A showing all the bands. Red boxes indicate the samples of interest. Figure S3. The uncropped Western blot images corresponding to Figure 7A showing all the bands. Red boxes indicate the samples of interest.
