# Supplementary material for: Pentagalloyl Glucose from Bouea macrophylla Suppresses the Epithelial–Mesenchymal Transition and Synergizes the Doxorubicin-Induced Anticancer and Anti-Migration Effects in Triple-Negative Breast Cancer
Source: Pharmaceuticals (Basel). 2024 Dec 20;17(12):1729. doi: 10.3390/ph17121729 (PMC11679756; doi:10.3390/ph17121729)
Supplement: Supplementary file 1 [file pharmaceuticals-17-01729-s001.zip › pharmaceuticals-3351365-supplementary.pdf]

Supplementary information

Article

Pentagalloyl Glucose from Bouea macrophylla Suppresses the Epithelial–Mesenchymal Transition and Synergizes the Doxorubicin-Induced Anticancer and Anti-Migration Effects in Triple-Negative Breast Cancer

Jiraporn Kantapan [1,2], Phattarawadee Innuan [1,2], Sarawut Kongkarnka [3], Padchanee Sangthong [4], Nathupakorn Dechsupa [1,2,*]

1. Molecular Imaging and Therapy Research Unit, Department of Radiologic Technology, Faculty of Associated Medical Sciences, Chiang Mai University, Chiang Mai 50200, Thailand; jiraporn.kan@cmu.ac.th; phattarawadeeinnuan@gmail.com, nathupakorn.d@cmu.ac.th
2. Department of Radiologic Technology, Faculty of Associated Medical Sciences, Chiang Mai University, Chiang Mai 50200, Thailand; jiraporn.kan@cmu.ac.th; phattarawadeeinnuan@gmail.com, nathupakorn.d@cmu.ac.th
3. Department of Pathology, Faculty of Medicine, Chiang Mai University, Chiang Mai 50200, Thailand; srawutzi@gmail.com
4. Department of Chemistry, Faculty of Science, Chiang Mai University, Chiang Mai, 50200, Thailand; padchanee.sangthong@cmu.ac.th

* Correspondence: nathupakorn.d@cmu.ac.th

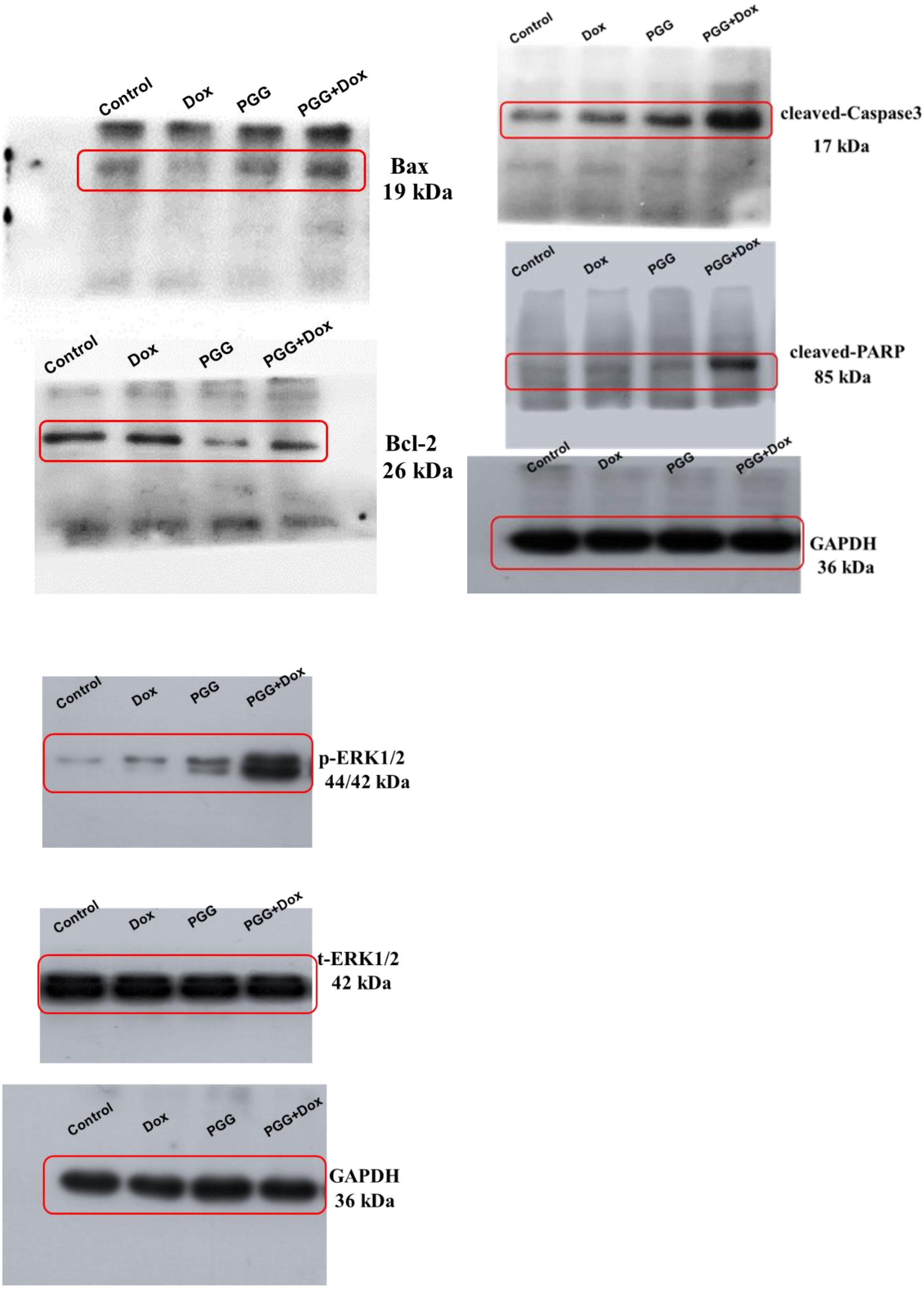

Supplementary Figure S1. The uncropped Western blot images corresponding to Figure 4E showing all the bands. Red boxes indicate the samples of interest.

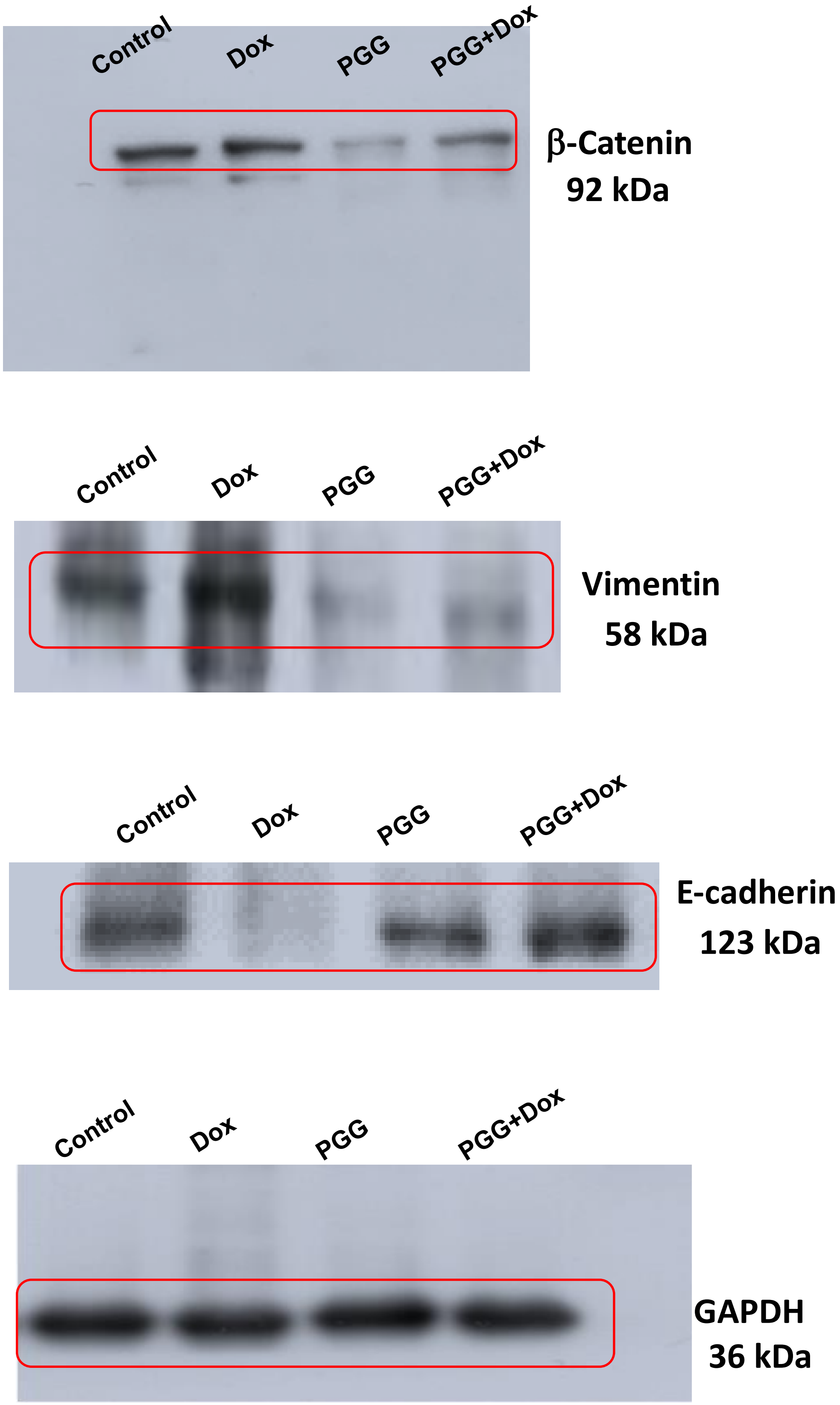

Supplementary Figure S2. The uncropped Western blot images corresponding to Figure 6A showing all the bands. Red boxes indicate the samples of interest.

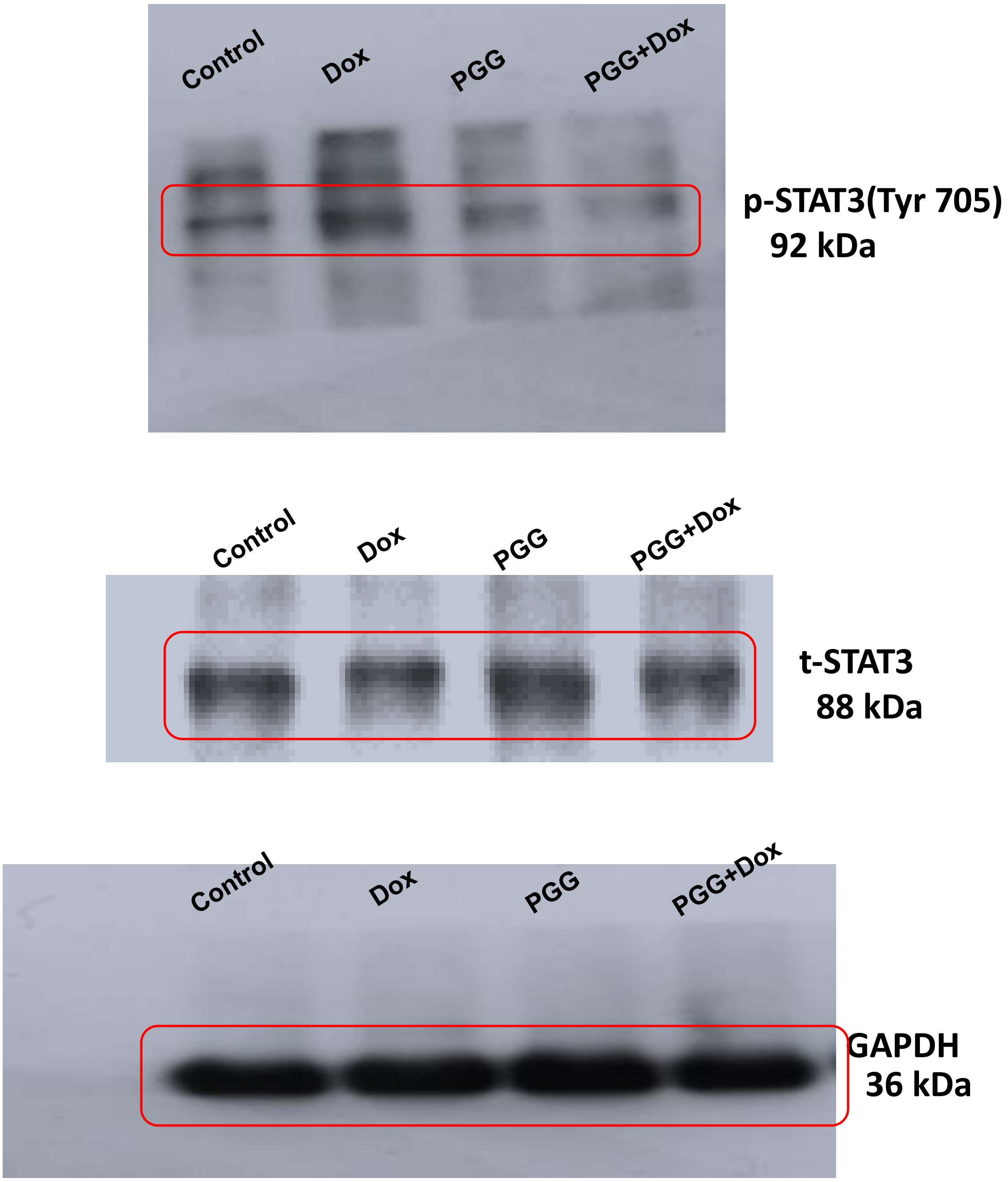

Supplementary Figure S3. The uncropped Western blot images corresponding to Figure 7A showing all the bands. Red boxes indicate the samples of interest.
